# Highlights from Heart Rhythm 2019: Atrial Fibrillation

**DOI:** 10.19102/icrm.2019.100805

**Published:** 2019-08-15

**Authors:** Rahul N. Doshi

**Affiliations:** ^1^Division of Cardiology, Keck School of Medicine, University of Southern California, Los Angeles, CA, USA

**Keywords:** Atrial fibrillation, commentary, Heart Rhythm Society, review

This year in San Francisco, the annual scientific sessions of the Heart Rhythm Society were not dominated by the buzz surrounding one landmark clinical trial [ie, Catheter Ablation versus Antiarrhythmic Drug Therapy for Atrial Fibrillation (AF) (CABANA); see last year’s commentary^1^]; instead, a multitude of aspects relating to the treatment of AF were front and center. In fact, eight of the 20 papers presented during the four late-breaking clinical trials sessions covered in some fashion the treatment of AF.

## Late-breaking clinical trials

Of note, two of the papers presented involved the use of autonomic modulation for the suppression of AF. The first was the Transcutaneous Electrical Vagus Nerve Stimulation to Suppress AF (TREAT AF) trial, presented by Dr. Stavros Stavrakis,^2^ which explored the use of tragal stimulation to decrease AF. This technique involves one-hour daily, low-level transcutaneous stimulation of the auricular branch of the vagal nerve, which was deployed in 53 patients with paroxysmal AF randomized to treatment or sham stimulation who were followed for six months. AF burden was assessed by ambulatory patch monitoring, and the stimulation provoked a significant 85% reduction in median AF burden (p = 0.011) at six months. The second trial was the Evaluate Renal Artery Denervation in Addition to Catheter Ablation to Eliminate AF (ERADICATE-AF) study presented by Dr. Jonathan Steinberg^3^ that explored the effects of renal artery denervation on AF when added to pulmonary vein (PV) isolation (PVI) over a 12-month study period. This was performed in five centers in Europe with patients randomized to either PVI with the cryoballoon plus radiofrequency ablation of the bilateral renal arteries or PVI alone. A total of 302 patients were randomized and followed for one year. At the end of the study period, 71.4% in the renal artery denervation plus PVI group were free of AF in comparison with 57.8% in the PVI-alone group (p = 0.011).

Taken together, both of the above studies present strategies that consider autonomic modulation in the treatment of AF, demonstrating the critical role that the autonomic nervous system has in the development and progression of AF. However, the quest for long-term effective strategies that target autonomics for other diseases such as hypertension or heart failure are more elusive. Perhaps lessons from another form of autonomic modulation are relevant—in this regard, Romanov and colleagues previously reported on the effects of the epicardial injection of botulinum toxin during open-heart surgery and the durability of benefit throughout three years of follow-up,^4^ despite elegantly demonstrating that the autonomic blockade as measured by heart rate variability returned to baseline at three months after treatment. As the authors suggested, perhaps AF is uniquely suited to benefit from effective autonomic modulation.

Similarly, two papers were presented examining the utility of “rotor” or wavefront-guided ablation in addition to standard catheter ablation. Dr. Johannes Brachmann presented the results of the Randomized Evaluation of AF Treatment with Focal Impulse and Rotor Modulation–guided Procedures (REAFFIRM) trial examining the effects of focal impulse or rotor modulation (FIRM)–guided mapping and ablation in patients with new-onset persistent AF.^5^ Three hundred seventy-five patients were randomized in a 1:1 fashion and followed for one year. While the addition of FIRM-guided ablation was safe, there was no demonstrated benefit upon combination with conventional ablation (69.3% for FIRM plus PVI versus 67.5% for PVI alone). The authors noted that the success rates in both arms were higher than those previously demonstrated in this population, perhaps relating to operator technique or experience. Similarly, Dr. Vivek Reddy presented the results of the Real-time Electrogram Analysis for Drivers of AF (RADAR) study, demonstrating the feasibility of the use of the RADAR system (AFTx Inc., Westminister, CO, USA).^6^ The system characterizes AF by organizing coronary sinus activation into phases and then identifies repetitive activity or drivers to target for ablation. In 64 patients (38 de novo and 26 redo procedures, respectively), the researchers demonstrated drivers throughout the left atrium and identified approximately a 70% single-procedure success rate at 12 months in their nonrandomized study cohort. The procedure was observed to be safe and attained higher success rates than those previously demonstrated in other trials for the ablation of persistent AF. The authors concluded that a randomized clinical trial should be performed to determine whether this technique is able to be added to PVI in persistent AF.

Taken together, these recent studies do not demonstrate any particular benefit at this time for incorporating an ablation strategy targeting rotors or drivers specific to AF in an individual patient but do perhaps suggest that approaches that include the ablation of additional areas beyond PVI in persistent AF may increase the overall efficacy of AF ablation. We clearly still have much to learn about how best to target these areas versus performing empiric anatomic ablation.

The potential for electroporation as an ablative technology in the treatment of AF has been met with much excitement, both with regard to its potential rapidity and its safety benefits. To this end, Dr. Reddy also presented follow-up data^7^ to the initial feasibility data presented last year at Heart Rhythm Society 2018. Considering two separate single-center cohorts with a combined total of 81 patients, the authors reported 100% efficacy was achieved for acute PVI with a single cardiac perforation. There were no occurrences of pulmonary stenosis, stroke or transient ischemic attack, magnetic resonance imaging lesions, phrenic nerve paralysis, esophageal lesions, or fistula formation. At 12 months, 87% of patients were free from AF. In patients who underwent redo procedures at three months, all 42 PVs remained electrically isolated. Electroporation as an energy source to create lesions is exciting due to its efficacy, though the primary driver of this technology is the safety profile. An additional report by Dr. Peter Loh using a different platform incorporating electroporation was also presented as a first-in-man study in 10 patients with an excellent initial safety profile and supportive evidence regarding potential safety benefits.^8^

## CABANA and heart failure

Given all the discussion that has happened regarding the outcomes of the CABANA trial, I would be remiss to leave out a critical presentation on the outcomes of the trial involving patients with heart failure (HF). Dr. Douglas Packer presented data from CABANA on the 778 patients with HF within the total patient cohort (n = 2,204).^9^ Patients in the trial with HF more often showed persistent AF as compared with non-HF patients and were more likely to be female or a minority and/or have hypertension, sleep apnea, or coronary artery disease. The primary outcome was a composite outcome of mortality, disabling stroke, serious bleeding, or cardiac arrest. Ablation as the primary treatment showed a statistically significant 36% reduction in the primary outcome as compared with drug therapy in the intention-to-treat analysis (p = 0.047). Similarly, total mortality was decreased by 47% (p = 0.034). There was additionally a 44% reduction in the likelihood of AF recurrence with ablation as compared with drug therapy over a four-year period, with a noted significant advantage in AF burden reduction.

Thus, despite the high crossover rate in the CABANA trial, in the intention-to-treat analysis, ablation was the superior therapy over drug therapy, reducing AF recurrence and burden and resulting in a significant benefit in the primary outcome as well as total mortality in patients with symptomatic HF.

## Other notable presentations

Dr. Arash Aryana presented on the long-term durability of posterior wall isolation using a cryoballoon approach for patients with persistent AF.^10^ In patients requiring a repeat ablation most commonly for atrial tachycardia, 88% experienced durable posterior wall isolation, and gaps were most commonly seen on the left atrial roof. Dr. Jorge Romero and colleagues also gave an important presentation regarding the risk of phrenic nerve damage during left atrial appendage electrical isolation and the importance of mapping location prior to ablation.^11^ The left phrenic nerve runs anterior to the left atrial appendage and its proximity to endocardial ablation sites varies, making safe isolation of the appendage difficult in some cases.

Dr. Rod Passman and colleagues presented findings from a large dataset constructed from an overlap of electronic health records and remote monitoring system data to characterize the relationship of CHA_2_DS_2_-VASc score combined with AF duration on thromboembolic risk.^12^ In a cohort of almost 22,000 patients, they identified a clear relationship regarding the risk of thromboembolism and AF duration as stratified by CHA_2_DS_2_-VASc score. Patients with a low or moderate risk for stroke with low or no AF burden had a low incidence of stroke off anticoagulation, while those with a borderline CHA_2_DS_2_-VASc score with a high AF burden were thought to likely benefit more so from oral anticoagulant therapy. The authors concluded that one should employ AF duration to further refine thromboembolic risk to more effectively stratify patients for oral anticoagulant therapy.

Lastly, in the area of AF diagnostics, Dr. Sarah Baalman and colleagues reported the use of a deep learning algorithm for single-beat discrimination of AF versus sinus rhythm,^13^ achieving a rate of 96% accuracy, which of course may have tremendous forthcoming applications in the age of wearable technology for AF detection.

Once again, the Annual Scientific Sessions of the Heart Rhythm Society did not disappoint in terms of content. I look forward to seeing everyone in San Diego!
